# Am. Med. Association, Section of Dental and Oral Surgery

**Published:** 1885-08

**Authors:** J. S. Marshall


THE
### AMERICAN JOURNAL
OF
### DENTAL SCIENCE

VOL. XIX. THIRD SERIES—AUGUST 1885. NO. 4.

ARTICLE I.

### AMERICAN MEDICAL ASSOCIATION.

### SECTION OF DENTAL AND ORAL SURGERY.

**Thirty-Sixth Annual Meeting, held at New Orleans, La., April 28, 1885.**

*Reported for the Dental Register, by Dr. J. S. Marshall.*

The General Meeting of the Association, though not as large as usual, was very interesting and profitable. Something over six hundred members were registered from all parts of the country, but this number would have been doubled had the place of meeting been more centrally located, or the time a month earlier. The weather was very warm and oppressive, which made the attendance upon the various sections somewhat less than it would have been under more favorable circumstances. The profession and citizens of New Orleans however did all in their power to make our stay among them enjoyable.

The local gatherings and receptions were particularly pleasant and were participated in very generally by the members of the Association and their lady friends.

The sessions of the Section on Dental and Oral Surgery were held in Grunewald Hall, each day, commencing at 3:30 P. M.

The section was called to order on Tuesday afternoon by Dr. J. S. Marshall, of Chicago, Ill., who stated that the officers of the section were both absent, Dr. W. W. Allport, Chairman, being detained at home through illness, and Dr. E. C. Briggs, the Secretary, by professional engagements. It would therefore be necessary to elect a Chairman and Secretary. On motion of Dr. A. E. Baldwin, of Chicago, Dr. Jacob S. Williams, of Boston, Mass., was unanimously chosen Chairman, pro tem.

Dr. Williams on taking the chair stated that the absence of the Secretary was entirely unavoidable. On motion of Geo. H. Fredericks, of New Orleans, Dr. John S. Marshall, of Chicago, was elected Secretary pro tem. The minutes of the last session of the section having already been published in the *Journal* of the Association the reading of them was dispensed with.

On motion of Dr. A. E. Baldwin, the privileges of the floor were extended to those gentleman present and practitioners of dental surgery who were not members of the Association.

Dr. John S. Marshall, of Chicago, Ill., then read a paper entitled:

### COCAINE IN DENTAL SURGERY.

The following is an abstract of the paper:

MR. CHAIRMAN AND GENTLEMEN:—The hydrochlorate of cocaine, like all new remedies which have given promise of mitigating the sufferings of mankind, was hailed with enthusiasm, and it is fast gaining a firm foothold in certain lines of practice as a local anesthetic, notably in operations upon the eye and upon mucous and serous tissues.

On the other hand, with the dental surgeons hydrochlorate of cocaine has lost much of its interest from the fact that it has proved a disappointment where it was hoped that it would be of the greatest benefit, viz., as an anesthetic or obtunder of sensitive dentine; and now that the enthusiasm over the drug has waned and we begin to investigate its claims with cooler heads and less biased judgment, many of the published accounts of its wonderful effects upon sensitive dentine, and other tissues of the teeth, it would seem must have originated very largely in the imaginations of the writers rather than that they were clinical facts. New forms of the drug, however, have been more recently introduced which give promise of better results, and my excuse for presenting a paper upon the subject of cocaine is to call your attention especially to the citrate of cocaine by presenting a series of experiments made with the hydrochlorate, the oleate, and citrate, and then leave you to judge which gives promise of being the most reliable local anesthetic or obtunder of sensitive dentine.

In operations upon the mucous membrane of the mouth there seems to be little choice between any of these forms, but upon sensitive dentine and pulp tissue it will be seen by the following experimental cases that the citrate is much more reliable than either of the others.

The cases selected were the first ten upon which each of these forms of cocaine were tried. *Hydrochlorate or muriate of cocaine*—C17 H21 NO4 H Cl=. With this form of cocaine I first used a 2 per cent. solution prepared by Foucar, but after several trials, more or less failures, I came to the conclusion that a dense tissue like dentine absorbs so slowly that a sufficient quantity of 2 per cent. solution could not be taken up to produce anesthesia, and therefore procured 10 per cent. and 20 per cent. solutions prepared by Merck, but was unable to obtain any better results with these than with the 2 per cent. A 40 per cent. solution has been recommended; this I have not tried, but from the experience of others who have experimented with it, it seems to have no advantage over the weaker solutions.

In cases Nos. 1 and 2 the rubber dam was not applied, and in case No. 4 of course there was no use for it; in all

### EXPERIMENTS WITH THE HYDROCHLORATE OF COCAINE—SUMMARY OF CASES.

| Case........... | Age........... | Structure of the Teeth. | Diseased Condition | Local Anesthetic. | Strength of Solution, per ct. | Number of Applications. | Immediate Effect......... | Time, Minutes. | Results. | Remarks. |
|---|---|---|---|---|---|---|---|---|---|---|
| 1 | 30 | Soft. | Sensitive Dentine. | Foucar's Sol. | 2 | 2 | None | 10 | Sensitiveness greatly relieved. | |
| 2 | 15 | Soft. | Sensitive dentine. | " | 2 | 4 | None | 20 | No anesthetic effect | |
| 3 | 24 | Medium. | Sensitive dentine. | " | 2 | 3 | None | 15 | No anesthetic effect. | |
| 4 | 24 | ......... | Pyorrhœa alveolaris. | " | 2 | 2 | None | 10 | Operated with little pain. Gums completely anesthetized. | A previous operation without the Cocaine had been very painful. |
| 5 | 50 | ......... | Inflamed pulp. | Merck's Sol. | 10 | 4 | None | 20 | No anesthetic effect. | Applied for extirpation. |
| 6 | 26 | Medium. | Sensitive dentine. | " | 10 | 3 | None | 15 | Slight anesthetic effect. | |
| 7 | 28 | Dense. | Sensitive dentine. | " | 10 | 3 | None | 15 | No anesthetic effect. | Drying the cavity with hot air seemed to produce a slight anesthetic effect. |
| 8 | 14 | Very Soft | Sensitive dentine. | " | 20 | 3 | None | 15 | Slight anesthetic effect. | Drying the cavity with hot air seemed to increase the anesthetic effect. |
| 9 | 16 | Medium. | Sensitive dentine. | " | 20 | 3 | None | 15 | Slight anesthetic effect. | Drying the cavity with hot air seemed to increase the anesthetic effect. |
| 10 | 24 | Medium. | Sensitive dentine. | " | 20 | 4 | None | 20 | No anesthetic effect. | |

### EXPERIMENTS WITH THE OLEATE OF COCAINE—SUMMARY OF CASES.

| Case | Age | Structure of the Teeth. | Diseased condition. | Local Anesthetic. McKesson & Robbins. | Strength of Oleate, per cent. | Number of Applications. | Immediate Effect | Time, Minutes. | Results. | Remarks. |
|---|---|---|---|---|---|---|---|---|---|---|
| 1 | 12 | ......... | Abscessed root. | Oleate. | 5 | 3 | None | 12 | Pain on extraction. | Gum anesthetic in 8 minutes. |
| 2 | 40 | ......... | Abscessed root. | " | 5 | 2 | None | 20 | Pain on extraction. | Gum anesthetic in 7 minutes. |
| 3 | 60 | Dense. | Inflamed pulp. | " | 5 | 3 | None | 30 | No anesthetic effect. | Applied normal oleate for 15 minutes, with no effect. Application made for extirpation. |
| 4 | 30 | Medium. | Sensitive dentine. | " | 5 | 3 | Slig't pain | 25 | No anesthetic effect. | |
| 5 | 25 | Soft. | Sensitive dentine. | " | 5 | 1 | None | 30 | No anesthetic effect. | Applied normal oleate for 15 minutes, with no effect. |
| 6 | 11 | Soft. | Sensitive dentine. | " | 5 | 3 | None | 20 | No anesthetic effect. | The hot air blast produced slight obtunding effect. |
| 7 | 14 | Medium. | Sensitive dentine. | " | 5 | 3 | None | 20 | Slight anesthetic effect. | |
| 8 | 40 | ......... | ......... | " | 5 | 2 | None | 30 | Gum lost sensation in 15 min. Root and border of alveolus quite sensitive. | Application made preparatory to fitting a gold band to the root of a central incisor sup. |
| | | | | " | 5 | 2 | | | | |
| 9 | 35 | ......... | Pyorrhœa alveolaris. | | | | None | 15 | Operated with little pain. | |
| 10 | 42 | Dense. | Sensitive dentine. | " | 5 | 2 | None | 20 | Slight anesthetic effect. | |

### EXPERIMENTS WITH THE CITRATE OF COCAINE—SUMMARY OF CASES.

| Case. | Age. | Structure of the Teeth. | Diseased Condition | Local anesthetic. McKesson & Robbins. | Amount of Citrate used, gr. | Number of Applications. | Immediate Effect. | Time, Minutes. | Results. | Remarks. |
|---|---|---|---|---|---|---|---|---|---|---|
| 1 | 26 | Soft. | Sensitive dentine. | Citrate. | 1–16 | 1 | Slight pain. | 10 | Complete anesthesia. | Lasted about one hour. |
| 2 | 19 | Medium. | Sensitive dentine. | " | 3–16 | 2 | Slight pain 5 min. | 15 | Complete anesthesia. | |
| 3 | 35 | Soft. | Sensitive dentine. | " | 5–16 | 3 | Severe pain 20 min. | 30 | Partial anesthesia. | Operation quite painful. |
| 4 | 20 | Medium. | Sensitive dentine. | " | 1–16 | 1 | Slight pain 3 min. | 10 | Complete anesthesia. | |
| 5 | 30 | ........ | Inflamed pulp. | " | 2–16 | 1 | None. | 15 | Complete anesthesia. | Extirpated the pulp without pain. |
| 6 | 16 | Soft. | Od'ntalgia | " | 7–16 | 3 | Patient suf. before citrate was applied. | 30<br>15 | No effect.<br>Complete anesthesia. | Patient relieved in 5 min. by the application of oil of cloves & sulp. morph. ⅛ gr. |
| 7 | 18 | Soft. | Sensitive dentine. | " | 2-16 | 2 | Slight pain 5 min. | 10 | Complete anesthesia. | |
| 8 | 24 | Dense. | Sensitive dentine. | " | 1–16 | 1 | Slight pain 3 min. | 30 | Partial anesthesia. | Operation slightly painful. |
| 9 | 23 | Soft. | Sensitive dentine. | " | 2–16 | 2 | Slight pain 5 min. | 10 | Complete anesthesia. | |
| 10 | 27 | Dense. | Sensitive dentine. | " | 1-16 | 1 | Slight pain 3 min. | | | |

others, after syringing the tooth with tepid water and removing all loose debris, the dam was adjusted, the cavity dried and the cocaine solution applied by a means of a pledget of cotton.

*Oleate of Cocaine.*—$C_{17}$ $H_{21}$ $NO_4$ $C_{18}$ $H_{33}$ $O_2$.

Normal oleate of cocaine contains from 48 per cent. to 52 per cent. alkaloid cocaine. Messrs. McKesson and Robbins kindly furnished me with samples of the normal and 5 per cent. oleates manufactured by them for this series of experiments.

In all cases of sensitive dentine, and the one with an exposed pulp, the rubber dam was adjusted and the cavity cleansed and dried, and the oleate applied on a pledget of cotton.

In the others the gums were dried and the oleate painted over the surface and the parts kept free from moisture during the operation by the use of napkins.

*Citrate of Cocaine.*—($C_{17}$ $H_{21}$ $NO_{42}$) $H_3$ $C_6$ $H_5$ $O_7$. This form of cocaine was first manufactured and recommended by Merck of Darmstadt, as likely to prove most satisfactory as an anesthetic for sensitive dentine.

My attention was first called to it by a letter in the *Journal* written from Weisbaden, December 4, 1884, by Dr. Sarah Hackett Stevenson.

I at once took steps to procure a sample of Merck's preparation, but, failing in this, Messrs. McKesson and Robbins kindly prepared a sample for me and this I had made into pills of one-fourth grain each.

The excipient used was gum tragacanth dissolved in glycerine. This readily dissolves on being moistened with tepid water and therefore makes a very convenient vehicle for the introduction of the citrate into the cavity. My method of using it is as follows: Remove all loose debris from the cavity, and wash it out with tepid water, then adjust the rubber dam, divide a pill into two or four equal parts and place one of these in the bottom of the cavity and cover it with a pledget of cotton moistened with tepid water.

The excipient soon dissolves and flows over the surface of the cavity. I five minutes I test the dentine and if still sensitlve make a second or third application if necessary.

Since recording the above cases I have had still further opportunity of testing the merits of the citrate of cocaine. In several of the cases of sensitive dentine where the hydrochlorate and the oleate failed I have since used the citrate with much better results.

From the cases recorded it will be noticed that the citrate is much more reliable as an anesthetic or obtunder of sensitive dentine than either the hydrochlorate or the oleate. Whether this is due to the special form of the drug or its greater concentration as used in the cavity I am unable to say.

It also seems to act much more promptly whether applied to sensitive dentine, pulp tissue, or mucous membrane.

Dr. Geo. H. Friedrichs, New Orleans. My son and I have used the hydrochlorate of cocaine in dental operations, but with no success.

The citrate we have not tried, because we have been unable to procure it.

From the report of the cases in which Dr. Marshall has used the drug, it seems to be much superior to the hydrochlorate.

The pain he speaks of as being caused by the application of the citrate to sensitive dentine, is caused, no doubt, by the abstraction of the fluids contained in the tubuli; and by this process the obtunding effect may be produced, just as it is by the use of the hot air blast, absolute alcohol, etc.

Dr. J. R. Walker, New Orleans. My experience with cocaine has been entirely with the four per cent. solution of the hydrochlorate, and it has not been very successful. I believe the general opinion of the profession to be that the hydrochlorate of cocaine is of little benefit in dental operations.

I have, however, succeeded in anæsthetizing one pulp and removing it with but little pain, and have extracted one tooth after applying the solution to the gums with a material decrease in the amount of pain usually suffered under such an operation. It has been recommended that if the solution were injected hypodermically into the gums, or in the region of the nerve trunk, that teeth might be extracted under its influence without pain.

Dr. Smith, Honolulu, Sandwich Islands. I have been practicing many years as a dentist and have been hoping that some remedy might be introduced that would be serviceable, and at the same time safe for the relief of the suffering often experienced by our patients while operating upon their teeth.

I have been delighted with the paper of Dr. Marshall upon cocaine.

I have not had an opportunity to test the metits of the drug, as I have for the present given up my profession, but from the cases reported by Dr. Marshall I should expect good results from it.

The essayist remarked that he finally devitalized with arsenic one of the pulps which he tried to anæsthetize with the hydrochlorate of cocaine.

The amount used (60 to 100 parts of a grain) is very small and could do no harm, but as generally used I have seen very bad results from it, and I always hesitate to apply it.

Dr. A. E. Baldwin, Chicago. I have had no experience with any other form of cocaine than the hydrochlorate, and my experience has been very much the same as that described in the cases reported by Dr. Marshall and the gentlemen who have just spoken. One experience I have had with cocaine which I will relate. In excavating a cavity under hydrochlorate, and failing to get the anæsthetic effect, yet feeling that something must be done to quiet the patient, I applied to the gum over the apex of the root a pellet of cotton saturated with sulphuric ether, and to my great astonishment I was able to finish the preparation of the cavity without pain. I have tried it upon cases since with as good results. With regard to arsenic I should say that the 100th part of a grain would be just as effective as a whole grain. All we want is the irritant effect, and that can be gained just as well with a small dose as with a large one. I am in the habit of applying dialized iron to the gum after the arsenic is in place as a precautionary measure in those cases where I find it necessary to resort to the use of arsenic for devitalization of the pulp. Referring again to the use of cocaine, I think the variability in the effect of the drug is due more to the result of the condition of the tooth and to the methods of applying it, than to any change in the drug itself. I should have been very glad if Dr. Marshall had described more minutely the extent of the caries, and pathological conditions of the cases upon which he experimented. It will be well for us to do this in our experiments with the drug.

Dr. Friedrichs. I think the ground just taken by the last speaker is entirety untenable. Cocaine is supposed to be a local anæsthetic. The pulp is not a nerve proper, neither are the nerve fibres of the dentine, they contain nothing but neuralemma; therefore the depth or extent of the caries would have no weight.

The shallow cavity is usually the most sensitive. Healthy pulps are not sensitive. This I had opportunity to demonstrate on one occasion when a boy was brought to me within a short time after receiving an injury which had split open a central incisor tooth, leaving the pulp fully exposed; this I could touch with a probe without causing the least pain.

Cocaine used at different times in the same case, does not always give the same results. In some individuals it has proved a success at one sitting, while at another it has been a failure.

Dr. Baldwin. The ideas advanced were not intended to be taken as facts, but simply as an opinion. I am surprised, however, to learn that a normal pulp is not sensitive.

When we excavate a cavity having a living pulp, the dentine is sensitive. Where does the dentine get its sensation, if not from the pulp? The pain must be transmitted by the pulp to the nerve centers, and if this is so the pulp must be sensitive. On mucous membrane cocaine must act by paralyzing the nerve fibres.

Dr. Walker. In all my experience I have never found any such condition of the pulp as described by Dr. Friedrichs. The pulps that I have seen exposed by traumatic lesions have always been sensitive. The sensitiveness is exhausted in certain pathological conditions and under various forms of irritation.

Dr. Marshall. In the case spoken of by Dr. Friedrichs it is not at all improbable that the pulp was paralyzed or rendered anæsthetic by the force of the blow, or its nerve fibres may have been ruptured at the apex of the root.

Dr. Thurber, New Orleans. I did not arrive early enough to hear the paper read by Dr. Marshall, and therefore cannot speak upon that. My experience is however very different from that of Dr. Friedrichs and others in the use of cocaine. I have used the hydrochlorate a good deal in operating upon the teeth of children and have been very much pleased with the results. I am in the habit of using the rubber dam to prevent the ingress of moisture and the consequent dilution of the solution. If all would use the rubber dam I think there would be less failures with the hydrochlorate of cocaine. I have been unsuccessful in extracting teeth by injecting the gums with the solution, but in extirpating pulps I have had better success. I consider cocaine a very valuable remedy as an obtunder of sensitive dentine and I should not be willing to do without it.

Dr. Williams. My experience with the hydrochlorate of cocaine has been like that of the majority of those who have discussed the paper. I have, however, seen the same failures in other remedies. Tannic acid, sulph. ether, aqua calcis, chloride of calcium, etc., in certain cases each of these will obtund the sensitiveness of dentine, but the failures occur more often than the successes.

With regard to the statement of Dr. Friedrichs that the normal pnlp is not sensitive, I am sorry to say that my experience does not agree with his. I once had occasion to open into a pulp which had not been previously irritated, but the tooth was slightly decayed, in this case I found the pulp quite sensitive.

Dr. Friedrichs. In the case just spoken of by Dr. Williams there was a lesion in the tooth, and consequently the pulp must have been to a greater or less extent in a pathological condition, while in my case there was no lesion of the tooth before the injury was received, and consequently the pulp was in a normal condition. The two cases are not alike in any particular.

Dr. Marshall closed the discussion by saying that he had used the hydrochlorate both with and without the exclusion of moisture from the cavity, but could see no difference in the effect. The successes and failures mentioned by Dr. Friedrichs, in his use of the hydrochlorate, as occurring in the same individual at different sittings, must have been the result of the decomposition of the solution.

For the benefit of those who would like to procure the citrate of cocaine for trial, I would say that McKesson & Robbins are now prepared to furnish the profession with the drug in one-eighth gram pills prepared at my suggestion. The price I cannot name, but that which I have been using cost from 80 cents to $1 per gram. It may be somewhat cheaper now.

Dr. Jacob L. Williams, of Boston, then read a paper entitled, "A Suggestion on the Proper Alternation of Rest with Efiort, as Essential to Health and Strength.

ABSTRACT.

MR. CHAIRMAN AND GENTLEMAN:

In my early pupilage I once received a very valuable suggestion from the venerable Dr. John C. Warren, long since deceased, to the effect, "When engaged in a long surgical operation of half an hour or more in duration, the eyes will sometimes become fatigued, and it will be difficult and unsafe to continue the operation with them in that condition. It is better under such circumstances to raise the eyes and let them rest on some object in a distant part of the room, or if you can do so leave the operation, step to the window and look out for a minute or two; you will then return with the eyes refreshed and you can see as well as ever."

More recently one of America's greatest ophthalmologists has written that one great cause of injury to vision is the continuous application of the eyes to study or work after they have become fatigued.

I refer to these opinions because they represent principles which hold good in the exercise of any faculty.

There is a common notion abroad that mere "exercise strengthens," and the other elements necessary to produce strength are lost sight of. Some seem to think that the longer and more vigorously they can exercise their faculties, the stronger they must be; as a result of this we see fatigue carried to exhaustion, and this is only another name for weakening or debility. Illustrations of this are common in all occupations and in all times of life.

Youth is often crowded with continuous study during the day, and many time it is carried into the night; as a result the mental powers become debilitated, sometimes permanently so.

The business or the professional man will not or cannot pause for rest, until he finds that his health has failed, and sometimes not even then, but drops at his post. The ambitious rower or pedestrian continues his efforts sometimes until his strength is gone or his constitution completely shattered. In our special department of practice, many labor too many hours continuously during the day, and perhaps add to this, extra work in the evening, till the nerves shake like so much loose cordage in the wind; and if the individual does not fall dead at his chair, as to my knowledge occurred in one instance at least, he finds a long period of rest needed to bring back a semblance of his former strength.

The essayist also called attention to the fact that we should not permit or subject our patients to the endurance of continuous suffering; such strain often requiring several days to recover from, and has sometimes been productive of serious results. He placed emphasis upon the term *continuous effort*, because from this comes the harm when carried beyond simple fatigue. Fatigue should never be carried to the point of exhaustion. This rule should be learned early in our professional life, viz., rest if possible, just when you are tired and let your patients do the same.

On account of the lateness of the hour the discussion of the paper of Dr. Williams was deferred till the Wednesday session.

On motion the session then adjourned.

WEDNESDAY'S SESSION.

The session was called to order by the chairman pro tem, Dr. Jacob L. Williams, of Boston, and at once took up the discussion of the paper read by Dr. Williams.

Dr. Walker. The advice given in the paper is very much needed by that part of the profession who practice dental surgery, but it is very difficult to heed. As a class we overtax our strength, and those who have a full practice break down early.

There are two classes of gentlemen whom these suggestions in the paper should warn, viz., those who from ambition to make money will not take the needed rest, and those who from force of circumstances think they cannot do so. These gentlemen are making a grave mistake, which sooner or later they will be forced by failure of health to acknowledge. This has been my own condition twice in my professional life, but I believe I have now learned wisdom. Without good health we cannot perform our best services, and consequently it is a duty to ourselves and to our patients to preverve the health which has been given us.

Dr. Baldwin. The subject is one that I cannot discuss from the standpoint of the dental surgeon, having so recently entered its ranks, but from my knowledge of the eye and my experience in the practice of medicine I can appreciate the force of the statements and suggestions made in the paper under discussion. I have found also that among the dentists of my acquaintance very many of them complain of the fatigue experienced after the operations. Rest is necessary if we would keep the mind and the body in perfect health. Rest of the eye is just as necessary as rest of the organs of the body after exercise, and if we fail to obey this law of nature, we must sooner or later pay the penalty of an outraged law.

Dr. Marshall. The suggestions in the paper now before us I consider of great value, especially to practitioners of dental surgery.

Those of us who have passed through like experiences with Dr. Walker can appreciate their value, and if those who have not passed through them would heed the advice, so eminently practical they may save themselves untold misery, for what misery is greater than the endurance of the tortures of a shattered nervous system. With regard to the eyes of the majority of dentists in full practice, I think I may safely say vision is not normal; few have perfect eyes. Many, very many, complain not only of the general fatigue after prolonged operations which tax the eye at a short focus, but of headache more or less severe, and this I believe is often caused by the strain put upon the eye at an unnatural focus. In a normal condition of the eye eighteen inches is about the reading distance, but, for various reasons in many nental operations, this had to be considerably shortened, sometimes to eight or ten inches.

Such a strain cannot but fatigue the eyes and sooner or later permanently injure them, and the suggestion to lift the eyes at frequent intervals and allow them to rest upon distant objects in the room, or out of doors, if heeded, will very materially assist in warding off the conditions that I have mentioned.

In my own case I was a great sufferer from headache for several years, and at last by accident discovered that my eyes were astigmatic. I at once had them examined by a competent ophthalmologist, who fitted me with glasses. This was four years ago, and since that, the attack of headache have diminished from three and sometimes four per week to one in a month or six weeks. I mention this fact for the benefit of others who may be afflicted as I have been.

Dr. Williams. Rest is something more than change of application. To fully recuperate there must be complete cessation of all work. Change of occupation, or application simply, does not help the case very much; nervous energy is still being used up. To rest the eyes they should not be simply lifted from one object and allowed to rest upon another; this is only change of application, but should be allowed to pass from one thing to another, or the lids closed and the eyes given complete repose.

Dr. Walker. I think change of occupation is just as much rest as is complete repose. In regard to the causes of the damage to our eyes, I think it may be accounted for in some cases by the bad arrangement of the light in the operating room. Light should never be permitted to come from two directions, or to be reflected back from a glaring wall. The walls should be tinted with some soft and neutral color so as to avoid a glaring light.

Dr. Baldwin. I think the range of the paper is greater than that which concerns ourselves. It takes into consideration also rest for our patients. They often have a nervous dread of our operations and are many times in a highly nervous condition from over work. In such cases I am in the habit of prescibing 15 to 20 grains of bromide of potassium from half an hour to an hour before operating and have obtained marked benefit from its use.

Dr. Smith. I think most of us are inclined to over-work our eyes. With the gold operator the strain upon them is great and almost constant for several hours every day; this of course must weary the eyes and tell upon them in a few years, but if there is added to this the effect that of cross lights, as just now suggested by Dr. Walker, they will sooner or later ruin the strongest eyes. With glasses we have but one focus and the eyes cannot get the rest so much needed while wearing them.

I think, however, the trouble is largely due to the effect of the cross lights. I am of the opinion the best place for the light is directly above, and have operated under such an arrangement of the light for years with the greatest satisfaction.

No class of professional men have so much trouble with their eyes as dentists. The close application at such short focus, as suggested by Dr. Marshall, is also an important fact to be considered, and I have no doubt but that it has much to do with bringing about the result just mentioned.

Dr. Williams closed the discussion by saying many dentists fail to secure the advantages to be derived from properly adjusted glasses for fear that it will be considered by their patients as an admission that their eyes are failing, and therefore not to be trusted longer with delicate operations. This is a great mistake. Engravers and watchmakers use artificial helps in their delicate work, and thus save their eyes.

Every dentist should have upon his operating table a large lens mounted with a handle for the examination of his operations, this would greatly lessen the strain otherwise placed upon the eyes.

The chairman announced the next paper on the programme was "Epulis Tumors," by Dr. T. W. Brophy, of Chicago, Ill.

Dr. Brophy not being present, the chairman called for the paper by Dr. Oscar J. Coskery, of Baltimore, Md.

ABSTRACT.

"A Case of Sarcoma of Lower Jaw with Successful Removal," by Oscar J. Coskery, M.D., Baltimore, Md.

Peter King, colored, aged 15, native of Maryland, was admitted into the City Hospital, Baltimore, on March, 31, 1882. Family history good. Personal history as follows: Between two and three years ago his attention was called to a generally enlarged condition of the left side of the lower jaw and swelling of the face. This gradually increased in size. The growth was recognized by his medical adviser to be confined to the inferior maxilla.

The tumor spread in every direction, the teeth were considerably displaced and the tongue crowded well over to the right side of the mouth. About two months previous to presenting himself an enlarged gland made its appearance in the left sub-maxillary triangle.

*Operation.*—April 15, 1882. The patient was placed under chloroform and an incision was made through the lower lip at the medium line and carried to a point just below the chin, then backwards to the angle of the jaw, passing over the most prominent portion of the tumor, and then upwards to the articulation. The flap was dissected off the tumor and turned up; at this point the facial artery, being cut, had to be ligated. The right central incisor was extracted and the jaw cut through with the metacarpal saw just at the right of the symphysis menti; the bone was then severed from its connections with the soft tissues of the floor of the mouth and strong traction made upon it, when the neck gave way. The bone forceps were applied and the head of the bone together with the remainder of the neck were wrenched from position. Hemorrhage, up to this point in the eperation, had not been very great, but upon cutting down upon the enlarged gland and enucleating it, very profuse venous bleeding came on, and it was found that a large branch of the external jugular had been cut, necessitating the application of ligatures at both ends. The flap was then placed in operation and an opening left for drainage at the angle of the jaw, and covered with dry lint and a bandage.

The boy did well from the first. To control the fetor of the discharge "Listerine" was used as a mouth wash. The flap united kindly and the patient left the hospital on May 6, 1882, twenty-one days after the operation, with only one suppurating point; that left open for drainage, and with very little deformity. The microscopic examination revealed the growth to be a "re-current fibro-sarcoma." The patient has been seen within a year, but there was no indication of a return of the disease.

Plaster models and photographs of the appearances of the face and jaw were exhibited together with the tumor and a micro-photograph of a section of the tumor.

DISCUSSION.

Dr. Marshall. Dr. Coskery states that when last seen about two years after the operation the patient gave no evidence of any recurrence of the disease; of course that is no guarantee that it will not recur, but it certainly gives hope that it may not. I would like to inquire if there had been any reproduction of the lost jaw.

Dr. Coskery. No sir! At least I am so informed by the physician who referred the case to me and who has had frequent opportunities to examine the patient.

Dr. Williams. Have you any statistics with regard to the frequency of such cases?

Dr. Coskery. I have no statistics, but this I can say: Very few cases of a like nature are on record. Many of the best works on surgery fail to notice the condition at all.

Dr. Baldwin. I am certainly pleased with the paper and grateful for the information Dr. Coskery has given us. I fear, however, that the disease will recur. Two years is not long enough time upon which to base an opinion that it will not recur.

The operation was certainly thorough, if we may judge from the specimen exhibited. It is better to go to the extreme of sacrificing considerable tissue than to save too much, for in this last direction the failures are most likely to occur.

Dr. Coskery. The criticism of Dr. Baldwin is just. The microscope revealed the tumor to be a recurrent fibroma, and that would indicate the disease as likely to return. I operated seven times in one case of this character that was located upon the breast, but my patient finally died.

Dr. Walker exhibited several casts of interesting cases of malpositions of the teeth and difficult cases of regulating which he had successfully treated.

The session then adjourned.

---

ARTICLE II.

